# Impact of Beliefs about Medicines on the Level of Intentional Non-Adherence to the Recommendations of Elderly Patients with Hypertension

**DOI:** 10.3390/ijerph18062825

**Published:** 2021-03-10

**Authors:** Natalia Świątoniowska-Lonc, Jacek Polański, Grzegorz Mazur, Beata Jankowska-Polańska

**Affiliations:** 1 Department of Clinical Nursing, Wroclaw Medical University, 51-618 Wrocław, Poland; bianko@poczta.onet.pl; 2 Department of Internal Medicine, Occupational Diseases, Hypertension and Clinical Oncology, Wroclaw Medical University, 50-556 Wrocław, Poland; polanoo@hotmail.com (J.P.); grzegorz.mazur@umed.wroc.pl (G.M.)

**Keywords:** beliefs, medication nonadherence, hypertension

## Abstract

Background: Non-adherence to pharmaceutical treatment is one of the most common causes of uncontrolled hypertension. Non-adherence may be intentional or unintentional. In the case of intentional non-adherence, it is crucial to understand the reasons behind it. The literature increasingly addresses the issue of beliefs and concerns about medication, but studies on this subject performed in a Polish population of hypertensive patients are still lacking. The aim of the study was to assess the level of intentional non-adherence among patients with hypertension, and to determine the relationship between beliefs about medication and the level of intentional non-adherence to treatment in elderly patients with hypertension. Material and methods: The study included 300 patients (106 of whom were male, mean age (SD) 71.71 (8.12) years) with hypertension, treated at a hypertension clinic. The following instruments were used: the Intentional Non-Adherence Scale (INAS) for evaluating intentional non-adherence, and the Beliefs about Medicines Questionnaire (BMQ) for evaluating patients’ beliefs and opinions regarding medication. Socio-demographic and clinical data were obtained from patients’ medical records. Results: The mean (SD) INAS score in the study was 47.28 (19.12). Patients were most concerned about the harm caused by medication, and least concerned about the necessity to take medication (mean score per item 3.49 vs. 2.14). Correlation analysis demonstrated weak correlations between BMQ and INAS: higher scores for necessity were associated with more intentional non-adherence (r = 0.174, *p* = 0.003), while higher scores for overuse, harm, and concerns were associated with less intentional non-adherence (respectively: r = −0.253, *p* < 0.001 vs. r = −0.336, *p* < 0.001 vs. r = −0.351, *p* < 0.001). In multiple-factor analysis, factors increasing the level of intentional non-adherence were elderly age (β = −0.352, *p* = 0.009), multimorbidity (β = −2.374, *p* = 0.035), and a higher BMQ concerns score (β = −1.376, *p* < 0.001), while being single was an independent predictor decreasing intentional non-adherence (β = 5.646, *p* = 0.013). Conclusions: The overall level of intentional non-adherence among patients with hypertension is moderate, but approximately one third of patients with hypertension demonstrate a high level of non-adherence. Independent determinants of intentional non-adherence include concerns, elderly age, multimorbidity, and being single.

## 1. Introduction

The World Health Organization (WHO) has identified non-adherence to treatment as one of the most serious problems in the treatment of both acute and chronic disease [1]. Non-adherence to the prescribed treatment protocol is a fundamental barrier to achieving the expected outcomes of evidence-based treatment. Research by the WHO indicates that on average, one in two patients no longer adheres to their treatment six months after starting it [2]. For patients with hypertension, the rate is 30–40% [3]. Despite the availability of effective medication to control hypertension, non-adherence to pharmaceutical treatment has been identified as one of the most common causes of uncontrolled hypertension [2].

Non-adherence is commonly observed and interferes with the achievement of intended treatment objectives, which in turn results in numerous rehospitalizations and an increased incidence of complications [4]. Non-adherence to treatment may be classified as intentional or unintentional [5]. Intentional non-adherence represents a patient’s active decision not to take their medication regularly or at all. Investigations into the causes of intentional non-adherence seem extremely important for improving treatment effectiveness and the conscious involvement of patients in the treatment process. A variety of factors have been proposed as causes of intentional non-adherence, including polypharmacy, complex therapeutic protocols, concerns about the adverse effects of medication, lack of belief in the possibility of recovery, feeling healthy and symptom-free, and others’ opinions about a specific medication [5,6,7,8,9]. Among patients with hypertension, the absence of symptoms is the most common cause of non-adherence [10]. The symptoms that do occur tend to be mild and do not significantly restrict the patient’s activity, which fosters the belief that regular treatment is unnecessary [10]. 

A multitude of factors affect treatment adherence. Negative perceptions of medication, manifesting in concerns about its ineffectiveness or adverse effects, are a significant impediment to adherence [7,11,12,13,14]. Patients’ specific medication beliefs may be categorized into two main areas: beliefs about the necessity of taking the prescribed medication to maintain one’s health now and in the future, and concerns about the potential negative effects of taking the medication (e.g., addiction or long-term adverse effects from regular use) [14,15]. Common beliefs about medicines concern their intrinsic nature, the degree to which they are perceived as essentially harmful, and the way they are administered by healthcare professionals. Some patients believe that medication helps them, while others believe that multiple harmful effects of the treatment may outweigh any positive outcomes. Some of the patients who have strong opinions about their medication avoid taking it or, conversely, abuse it. One cause of intentional non-adherence may involve perceiving the medication as a reminder of one’s disease, which represents a threat to the patient’s identity [16]. Najjuma et al. in their study conducted at an out-patient clinic of a regional referral hospital in southwestern Uganda with hypertension showed that stigma due to the disease is one of the barriers to adherence to hypotensive medication [16]. Moreover, patients convinced that a specific medication is necessary for their health are much more likely to adhere to the treatment than those who do not hold such a belief [11]. Different levels of willingness and ability to follow pharmaceutical recommendations are observed particularly among elderly patients with chronic disease and multi-drug problems. It is highly probable that their attitude towards the treatment, their convictions and fears may significantly influence the level of adherence to the established therapeutic protocol [12,13]. Older people may have developed beliefs and views about the drugs they use, often based on their own or their family’s previous experience. Particularly suggestible patients are especially likely to develop preconceptions about medication. They may obtain information about medication, e.g., from the media, health reports, or social media sites [17]. In the case of patients with polypharmacy, the risk of side effects increases, and patients may have habits of medication abuse or believe that the treatment is of little benefit to them and may even be harmful.

Though the number of recognized factors affecting adherence is increasing, the literature indicates that many of these factors remain poorly understood [14,15]. Even with regard to intentional non-adherence, the focus is still placed on the negative aspects of treatment, such as adverse reactions, which may cause patients to be concerned about taking the prescribed medication. An understanding of the mechanisms behind such beliefs and their association with adherence seems extremely important for clinical practice. However, few publications focus on assessing beliefs about medication or the link between beliefs and non-adherence. Studies on the causes of intentional non-adherence remain scarce. Authors with an interest in adherence report a need for further studies that would evaluate the impact of beliefs about medication on adherence to treatment in chronically ill patients. To the best of our knowledge, this is the first Polish study, beyond the validation of the BMQ questionnaire, focusing on the relationship between beliefs about medication and adherence to treatment in patients with hypertension. Therefore, the aim of our study was to assess the level of intentional non-adherence among patients with hypertension, and to determine the relationship between beliefs about medication and the level of intentional non-adherence to treatment in elderly patients with hypertension.

## 2. Materials and Methods

### 2.1. Study Design and Setting

The present study has a cross-sectional, observational design. The study used closed-ended standardized surveys.

Patients with a clinically confirmed hypertension diagnosis, hospitalized in an internal medicine department, were recruited. Patients admitted to the department for diagnostic tests to follow up on the previously planned hypertension treatment were recruited for the study on their first day of hospitalization. Inclusion criteria were as follows: a clinical diagnosis of hypertension as per the ESC guidelines [18], age ≥18 years, chronic treatment with at least 1 antihypertensive drug for at least 6 months, and written informed consent to participate in the study. Exclusion criteria were as follows: exacerbation of another chronic condition (chronic heart failure—NYHA-IV (New York Heart Association Scale), ischemic heart disease—CCS-IV (Canadian Cardiovascular Society Scale), neoplastic disease, acute respiratory disease), inability to complete the questionnaire, or lack of written informed consent to participate. Elderly patients were assessed using the MMSE (Mini-Mental State Examination) questionnaire. Those with MMSE scores of ≤18 points [19] were excluded from the study.

In the study period (August 2019–January 2020), 396 patients with hypertension in accordance with the ESC criteria were hospitalized in the department [18]. In this group, 62 patients did not meet the inclusion criteria, and 14 refused to participate. Therefore, 320 patients were included in the study and received surveys; however, during the study, 20 patients dropped out without providing a reason or did not complete the survey correctly. Thus, the final study group included 300 patients diagnosed with hypertension and who were receiving pharmaceutical treatment. All patients were informed about the study course and methods, and about the possibility of withdrawing from the study at any time. All patients provided their written informed consent to participate in the anonymous survey.

Patients admitted to the department for diagnostic tests to follow up on the previously planned antihypertensive treatment were recruited for the study on their first day of hospitalization. The study was performed by a properly trained team including a physician specializing in hypertension and two internal medicine nurses responsible for cognitive function assessment and survey distribution. All team members received the study protocol so that they could collect data in the same way. Patients completed the questionnaires themselves, based on their last 4 weeks of treatment. Socio-demographic and clinical data were obtained from hospital records, with the patients’ consent. 

### 2.2. Questionnaires

The Intentional Non-Adherence Scale (INAS) is used to identify intentional non-adherence. It is a 22-item scale scored on a 5-point Likert scale (1 = strongly disagree, 5 = strongly agree). The total score ranges between 22 and 110, and higher scores indicate poorer adherence. In our study, patients were broken down into groups based on their INAS scores: group I—low intentional non-adherence (<55 points, *n* = 217), group II—high intentional non-adherence (≥55 points, *n* = 83). The questionnaire has very good psychometric properties. For the Resisting Illness (RI) scale, the Cronbach alpha co-efficient was 0.95, and for the Testing treatment (TT) scale, the alpha was 0.93 [11]. The use of the questionnaire was approved by John Weinman [15].

The Beliefs about Medicines Questionnaire (BMQ) allows for the assessment of the respondent’s beliefs regarding four aspects of medication use: overuse, harm, necessity, and concerns. The BMQ is a 10-item questionnaire scored on a 5-point Likert scale, identifying general attitudes and beliefs toward medicines, the necessity of taking the medication, and the level of concern about the medication the patient is currently taking (1 = strongly agree, 5 = strongly disagree). Higher scores indicate stronger beliefs [7,14].

### 2.3. Ethical Consideration

The study was approved by the Bioethics Committee of Wroclaw Medical University (approval no. KB 42/2019). Participation was voluntary and anonymous, and all patients were informed about the purpose, methods, and course of the study, and about their right to decline or discontinue their participation. Written informed consent was obtained from each participant prior to their inclusion, and the investigation conformed to the principles outlined in the Declaration of Helsinki.

### 2.4. Statistical Methods

Comparisons of qualitative variables in groups were conducted with chi-squared test (with Yates’ correction for 2 × 2 tables) or with Fisher’s exact test (when low expected values occurred). Comparisons of quantitative variables in two groups were conducted with the Mann–Whitney test. Correlations between quantitative variables were assessed with Spearman’s correlation coefficient. Multivariate analysis of simultaneous impact of many independent variables on one quantitative dependent variable was made by the means of linear regression. The 95% confidence intervals were reported along with regression parameters. Analyses were conducted at 0.05 level of significance. R software (Vienna, Austria), version 4.0.1 was used [20].

## 3. Results

### 3.1. Socio-Demographic and Clinical Characteristics of the Study Group

The study included 300 patients (mean (SD) age 71.71 (8.12) years), of whom 64.67% were female. 

Most patients had completed secondary education (45.67%) and were married (62.67%)—Table 1. The patients had a mean (SD) of 1.88 (0.94) comorbidities besides hypertension and took a mean (SD) of 2.12 (1.03) antihypertensive drugs. A total of 41.67% of the respondents were overweight. The mean (SD) blood pressure in the group was as follows: SBP (systolic blood pressure)—141.11 (40.15), DBP (diastolic blood pressure)—81.38 (7.4). 

### 3.2. Level of Non-Adherence (INAS Scores) and Beliefs about Medication (BMQ Scores)

The mean (SD)total INAS score was 47.28 (19.12) points (Table 2). A total of 72.33% of patients had a low and 27.67% a high level of intentional non-adherence. The patients obtained moderate scores on the overuse (mean = 11.92; SD = 2.67), harm (mean = 13.96; SD = 2.84), necessity (mean = 10.68; SD = 2.14), and concerns (mean = 15.50; SD = 3.10) subscales of the BMQ. They were most concerned about the harm caused by medication, and least concerned about the necessity to take medication (mean score per item 3.49 vs. 2.14).

### 3.3. Correlation Analysis for Non-Adherence and Beliefs about Medication 

Our analysis of correlations between beliefs about medication (BMQ) and non-adherence (INAS) demonstrated that all BMQ domains were significantly correlated with INAS scores (*p* < 0.05).

**Figure 1:**
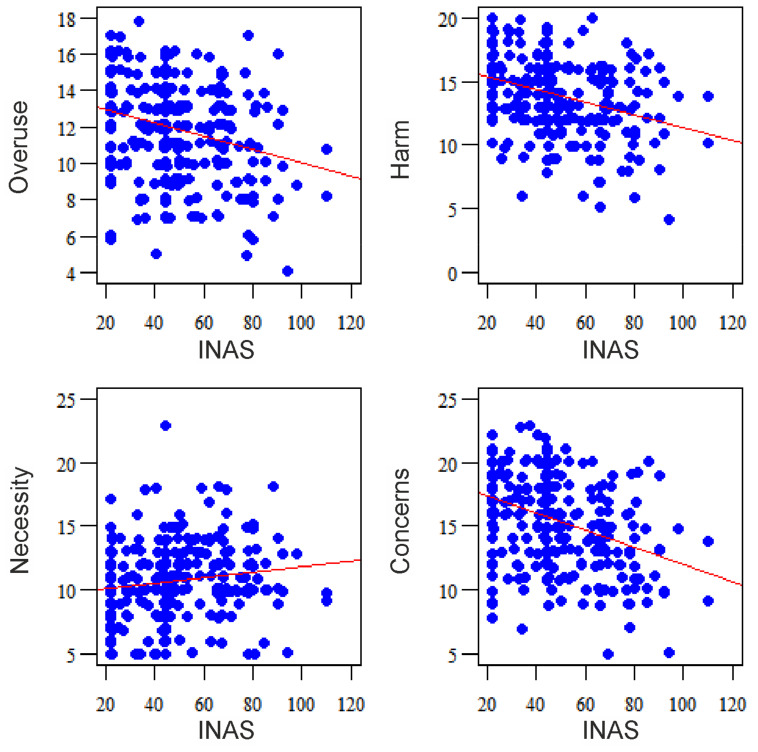
Correlation of BMQ domains with INAS.

In the correlation analysis, higher scores for necessity were associated with more non-adherence to treatment (r = 0.174, *p* = 0.003), while higher scores for overuse, harm, and concerns were associated with less intentional non-adherence (respectively: r = −0.253, *p* < 0.001 vs. r = −0.336, *p* < 0.001 vs. r = −0.351, *p* < 0.001) (Figure 1). These associations are bidirectional, i.e., higher INAS scores are associated with higher scores for necessity and lower scores for overuse, harm, and concerns.

### 3.4. Predictors of Intentional Non-Adherence

Our multiple-factor linear regression model showed that independent determinants of higher non-adherence levels include elderly age (β = −0.352, *p* = 0.009), more comorbidities (β = −2.374, *p* = 0.035), and a higher BMQ concerns score (β = −1.376, *p* < 0.001) (Table 3). Being single is an independent predictor of lower intentional non-adherence levels (β = 5.646, *p* = 0.013).

The R^2^ coefficient for the model is 23.53%, meaning that variables included in the model account for 23.53% of variance in the INAS scores. The remaining 76.47% depends on variables not included in the model or random factors.

## 4. Discussion

The aim of our study was to assess the level of intentional non-adherence among patients with hypertension, and to determine the relationship between beliefs about medication and the level of intentional non-adherence to treatment in elderly patients with hypertension. To the best of our knowledge, it is the first Polish study, excluding a validation of the BMQ questionnaire, concerning beliefs about medication as a determinant of adherence to treatment in a population of Polish patients with hypertension. The use of a standardized questionnaire for intentional non-adherence assessment is a strength of our study. 

Non-adherence is a common occurrence, and it prevents the patient from achieving the envisioned treatment benefits. The patient group studied demonstrated varying non-adherence levels. One third of the patients demonstrated high levels of intentional non-adherence to treatment. A similar result (27.9%) was reported in the study by Bae et al. in a group of patients with hypertension [21]. However, the literature indicates that most patients fail to adhere to treatment, either intentionally or unintentionally [21,22,23]. Some researchers suggest that during occasional episodes of unintentional non-adherence, patients may be testing the effectiveness of their medication or the severity of symptoms when untreated [24,25]. Some patients think that forgetfulness or carelessness are considered more socially acceptable as reasons for non-adherence than a deliberate decision not to take medication [26]. 

The literature also addresses the issue of patients’ concerns about their medication, which do affect their adherence to the prescribed treatment protocols or the regularity of medication-taking [8,9,23,27,28,29,30,31,32,33]. According to the Necessity-Concerns Framework, perceived personal need to take medication for current and future health (beliefs of necessity) and concerns about potential negative consequences are key beliefs that influence medication adherence [27]. Patients often need to balance their feelings about the necessity to take medication and the related concerns [5,7,15]. Patients who are indifferent to the prescribed medication have no considerable concerns about it, but are similarly unconvinced about the necessity of taking it [27]. In the present study, concerns were a significant determinant of intentional non-adherence. According to Náfrádi et al, patients who occasionally manifested intentional non-adherence behaviors reported stronger concern beliefs [33]. Similarly, in a study by Leporini et al. in an elderly group, inpatients, outpatients, and care facility residents were all concerned about the adverse effects of medication, which represented the main reason for non-adherence [29]. As reported by Glombiewski, concerns about the medication’s adverse effects are the most common cause of non-adherence [8]. In Clyne et al., most patients believed strongly in the necessity of taking their medication, while a third reported strong concerns about the drugs they took [30]. Horne and Weinman showed that patients who had fewer concerns about the side effects of their medication and a stronger belief in the necessity of taking it demonstrated better adherence [14]. Ratcliffe et al. reported that patients aged 60 and above are more concerned about the risk of relatively rare and severe adverse events than about those that are common and referred to as mild adverse reactions [9]. Notably, according to Modig et al., the benefits of treatment outweigh any potential adverse effects for most elderly patients [31,32]. 

Due to a lack of sufficient knowledge, patients who have no symptoms and find out about their hypertension by measuring their blood pressure do not adhere to treatment properly [10]. Patients who do not see the immediate effects of treatment, which are visible to them through the alleviation or elimination of ailments, do not feel the need to take medication to prevent cardiovascular events or other complications. Patients tend not to believe in the necessity of taking medication intended to prolong their life or contribute to secondary prevention of disease, rather than to simply alleviate symptoms [30]. In the Clyne study, some participants were concerned about long-term use of medications and potential adverse side effects, especially for medications prescribed to prevent future problems [30]. In contrast, drugs prescribed to alleviate symptoms were seen as beneficial and more readily accepted, often despite potential side effects [30]. As patients with hypertension may experience no symptoms, they may fail to recognize the necessity of taking antihypertensive medication [10]. In our study, most patients were unconvinced about the necessity of taking their medication. As the theoretical framework of Horne and Weinman suggests, pharmacological adherence decisions of chronically ill patients are influenced by an assessment of the benefits of treatment, where personal beliefs about the need for a drug to maintain or improve health are balanced by concerns about potential adverse drug reactions [27]. One unanticipated finding was the high score for necessity in patients with high levels of intentional non-adherence to treatment, which, however, was not identified as a significant determinant of intentional non-adherence. Schüz et al. demonstrated that increases in specific necessity beliefs predicted improved intentional adherence to medication, while the belief that a medication was harmful predicted a greater likelihood of intentional non-adherence [34]. Some researchers claim that factors considered to be determinants of intentional non-adherence may also cause unintentional non-adherence [23,28]. In addition, unintentional non-adherence may precede intentional non-adherence [23]. In the Gadkari and McHorney study, there was the direct, significant effect of unintentional non-adherence on intentional non-adherence [23].

In our study, in addition to the impact of beliefs about medication, regression analysis demonstrated that elderly age, being single, and multimorbidity were negatively correlated with intentional non-adherence levels. There is an ongoing discussion in the literature regarding links between age and adherence. According to some available publications, younger patients have higher unintentional non-adherence levels, which are associated with their lifestyle and professional activity [35,36]. However, there are also publications reporting an adverse impact of elderly age or the associated cognitive impairment or frailty syndrome on adherence, resulting in higher levels of unintentional non-adherence to treatment [37,38]. On the other hand, older patients may devote more time to adhering to pharmaceutical treatment. In addition, they are more likely to use devices facilitating regular medication-taking, such as pillboxes or calendars. 

Another predictor of lower intentional non-adherence levels in our study was being single, which is more commonly reported as a determinant of unintentional non-adherence, associated with a lack of motivation to follow treatment recommendations [39]. 

Multimorbidity was another significant clinical predictor of intentional non-adherence in our regression analysis. Multimorbidity has a direct impact on the number of medications taken. Elderly patients affected by polypharmacy are more likely to experience adverse effects and drug interactions, which may affect their concerns about medication [3]. Grant et al. demonstrated that elderly patients with polypharmacy were more likely to forgo medications with immediate effects, compared to those producing long-term benefits [40]. Evidence suggests that patients make selective decisions about non-adherence when prescribed multiple drugs. A study in a group of patients with type 2 diabetes showed that those with suboptimal overall adherence usually had problems with a single specific drug in their treatment regimen [40]. Patients taking threeor more medications tended not to adhere to only one of these medications [40]. Such intentional behaviors may indicate a patient’s attempt to avoid the adverse effects of the prescribed drugs. However, the role of age, relationship status, and treatment protocol complexity in intentional non-adherence to pharmaceutical treatment in elderly patients has not yet been fully understood.

There is a strong need to identify and assess the beliefs, preferences, and concerns of elderly patients in order to plan effective strategies to improve treatment efficacy. Routine evaluation of patients’ beliefs about medicines, adherence to treatment, and reasons behind any lack of acceptance for the recommended treatment should be introduced in family medicine practice [41]. Adherence and factors interfering with adherence should be routinely evaluated in daily clinical practice. An understanding of mechanisms behind intentional non-adherence, beliefs about medication, and concerns regarding the prescribed medication will facilitate the implementation of educational programs to improve treatment outcomes by shaping positive patient attitudes toward the treatment process.

Our study had a number of limitations. Non-adherence to treatment was assessed based on patients’ responses to questions in the questionnaire and their blood pressure. No objective measures of non-adherence were used, such as tests for medication concentrations in the blood, prescription fill rates, or medication purchased/taken. Additionally, we did not consider unintentional non-adherence levels. The overall non-adherence rate could be higher if unintentional non-adherence were included. However, the questionnaire we used to assess non-adherence has satisfactory psychometric properties and is commonly used for intentional non-adherence testing. Another limitation was due to the inclusion of patients from a single center. The study was also limited by the lack of psychosocial determinant evaluation (religion, faith, family support or assistance, and depression), while the latter disorder has been reported in the literature as a potential contributor to non-adherence.

## 5. Conclusions

1. The overall level of intentional non-adherence among patients with hypertension is moderate, but approximately one third of patients with hypertension demonstrate a high level of non-adherence.

2. In terms of beliefs about medication, patients with hypertension demonstrate high levels of beliefs about harm and concerns. However, in regression analysis, only concerns were an independent determinant of non-adherence.

3. Other independent determinants of intentional non-adherence included elderly age, multimorbidity, and being single.

## Figures and Tables

**Table 1:** Sociodemographic and clinical characteristics of patients according to INAS.

Variable		Total (*N* = 300)
Age [years]	Mean (SD)	71.71 (8.12)
Gender	Male	106 (35.33%)
	Female	194 (64.67%)
Education	None or primary school	130 (43.33%)
	Secondary school	137 (45.67%)
	University	33 (11.00%)
Marital status	Married or living with partner	188 (62.67%)
	Single or divorced	112 (37.33%)
BMI	Normal	40 (13.33%)
	Overweight	125 (41.67%)
	Obesity	95 (31.67%)
	Obesity class II	25 (8.33%)
	Obesity class III	15 (5.00%)
SBP [mmHg]	Mean (SD)	141.11(40.15)
DBP [mmHg]	Mean (SD)	81.38 (7.4)
Number of chronic diseases	Mean (SD)	1.88 (0.94)
Total number of medications taken	Mean (SD)	1.97 (1.96)
Number of hypertensive drugs	Mean (SD)	2.12 (1.03)

SD—standard deviation, SBP—systolic blood pressure, DBP—diastolic blood pressure, BMI—Body Mass Index, INAS—the Intentional Non-Adherence Scale, BMQ—the Beliefs about Medicines Questionnaire.

**Table 2:** INAS and BMQ questionnaire results.

Questionnaire		*N*	Range	Mean	SD	Mean per Question
INAS		300	22–110	47.28	19.12	-
BMQ	Overuse	300	4–20	11.92	2.67	2.98
	Harm	300	4–20	13.96	2.84	3.49
	Necessity	300	5–25	10.68	2.88	2.14
	Concerns	300	5–25	15.50	3.54	3.10

SD—standard deviation, INAS—the Intentional Non-Adherence Scale, BMQ—the Beliefs about Medicines Questionnaire, *N*—number of patients.

**Table 3:** Results of linear regression analysis.

Variable		Parameter	95% CI		*p*
Gender	Male	ref.			
	Female	−1.134	−5.396	3.129	0.603
Age	[years]	−0.352	−0.614	−0.09	0.009 *
Education	None or primary school	ref.			
	Secondary school	2.194	−2.291	6.679	0.339
	University	−1.419	−8.594	5.755	0.699
Marital status	Married or living with partner	ref.			
	Single or divorced	5.646	1.201	10.092	0.013 *
SBP	[mmHg]	0.038	−0.013	0.089	0.145
DBP	[mmHg]	−0.154	−0.436	0.128	0.287
Number of chronic diseases		−2.374	−4.565	−0.183	0.035 *
Number of hypertensive drugs		0.413	−1.581	2.408	0.685
MMSE		−0.36	−0.932	0.212	0.218
BMI	Normal	ref.			
	Overweight	3.116	−3.295	9.528	0.342
	Obesity	1.567	−5.005	8.14	0.641
BMQ	Overuse	−0.091	−1.114	0.932	0.862
	Harm	−0.88	−1.957	0.197	0.111
	Necessity	0.472	−0.294	1.238	0.228
	Concerns	−1.376	−2.097	−0.655	<0.001*

*p*—multivariate linear regression, BMQ—the Beliefs about Medicines Questionnaire, SBP—systolic blood pressure, DBP—diastolic blood pressure, MMSE—Mini-Mental State Examination, BMI—Body Mass Index, INAS—the Intentional Non-Adherence Scale, *statistically significant (*p* < 0.05).

## Data Availability

The data are not publicly available due to privacy and ethical restrictions. The data presented in this study may be available conditionally from the corresponding author.
